# Peony diagram modeling for improving the easy disassembly design of mobile electronic products

**DOI:** 10.1038/s41598-025-85402-7

**Published:** 2025-01-08

**Authors:** Junling Huang, Chuangchuang Cui, Wei Jiang, Libin Zhu

**Affiliations:** 1 School of Mechanical Engineering, Anhui Institute of Information Technology, Wuhu, 241000 China; 2 School of Mechanical Engineering, Hefei University of Technology, Hefei, 230009 China; 3 ULTIMATE Transportation Technology Co. Ltd., Qingdao, 266101 China

**Keywords:** Evaluate disassembly difficulty, Key components, Visual disassembly information model, Smartphones, Mechanical engineering, Sustainability

## Abstract

Designing mobile electronic products for easy disassembly is crucial for promoting resource recycling. However, many current approaches overlook the need to consider the disassembly of parts from the perspective of the overall product structure and practical recycling requirements, leading to potentially suboptimal or unnecessary optimization strategies. This study introduces a novel visual disassembly information model called the “Peony Diagram.” This model uses a multi-layer ring diagram to depict part hierarchies and constraint relationships and employs specific symbols to convey part disassembly information and highlight key components. The difficulty of disassembling individual parts is assessed by calculating difficulty coefficients based on quantified disassembly information in the model. To validate the model’s effectiveness, a smartphone was used as a case study to determine the shortest disassembly sequence and evaluate disassembly difficulty for both single and multiple parts. The findings demonstrate that the proposed method can accurately assess the ease of disassembly of key components during product repair and recycling. Consequently, it can suggest strategies to enhance recycling and disassembly efficiency and reduce the difficulty of disassembling critical parts.

## Introduction

E-waste is one of the fastest-growing waste streams globally, increasing by approximately 2 million tons annually, and is projected to reach 74.7 million tons by 2030, and discarded mobile electronic equipment constitutes a significant portion of this waste stream^1^. The rapid pace of technological advancement has shortened the upgrade cycle of smartphones to roughly one to two years^2^, contributing to a rise in electronic waste. Assessing the ease of disassembly of products is crucial for effective design and recycling practices. The European Commission has undertaken a study on the eco-design and energy labeling readiness of cell phones, smartphones, and tablets^3^. These initiatives are steering the industry towards producing more environmentally friendly and recyclable products^4^. To facilitate a smooth and efficient transition, it is essential to evaluate the disassembly ease of the next generation of products. Such assessments help identify and resolve potential disassembly challenges, ensuring that design improvements genuinely foster resource recycling and sustainability throughout the life cycle of electronic products.

In recent years, significant research has been conducted on assessing the ease of product disassembly. Aslain Brisco Ngnassi Djami (2020)^5^ and Jessicade Aguiar and Luana de Oliveira (2017)^6^ have emphasized the importance of evaluating product disassemblability at the early stages of design. Ellen Bracquené^7^ explored methods for assessing product reparability during the use phase to extend product life. Studies by S. Harivardhini (2016)^8^ and Houda Bouyarmane (2020)^9^ have focused on evaluating the ease of disassembly at the end-of-life (EoL) stage. Although these studies contribute significantly to their respective fields, they often fail to prioritize the disassembly of key components during the actual disassembly process. Not all parts of a product need to be disassembled in practice. Partial disassembly is more efficient for removing hazardous parts and accessing valuable components compared to complete disassembly^10^. Wang K noted that dismantling firms often maximize profits through partial dismantling^11^. The EN45554:2020 standard introduces a methodology for assessing the repairability, reusability, and upgradability of relevant products, highlighting the need to identify and prioritize “priority components^12^”. Desai, A. (2020) proposed the hotspot mapping method, which indicates the difficulty of disassembling key components by labeling hotspots in a Table^13^. Previous research methods have primarily focused on calculating and evaluating the ease of disassembly of product components. To further enhance the easy-disassembly design of products, it is necessary to develop a new method that comprehensively considers various factors in the design process, thereby improving the overall easy-disassembly performance of products.

The objective of this study is to develop a visualization model to enhance the easy disassembly design of a product, thereby providing decision support and references for designers to improve the disassembly design of key product components. This study introduces an innovative visual disassembly model named the “Peony Diagram,” which visualizes product component layouts and disassembly information. The diagram reflects the disassembly level, constraints, and shortest disassembly paths for each component. Additionally, the model’s disassembly information helps calculate part disassembly difficulty, providing a crucial reference for optimizing easy-to-disassemble designs during the design phase.

Section “Literature review” reviews existing modeling approaches and methods for assessing ease of disassembly. Section “Methods” outlines the proposed modeling approach and the method for quantifying disassembly difficulty used in this study. Section “Case study” presents case applications, where two smartphones are used as examples to model and validate the shortest disassembly sequence and the disassembly difficulty of components. Section “Discussion” provides a discussion and proposes optimizations based on the case results. Finally, section “Conclusions” summarizes the contributions and limitations of the study.

##  Literature review

This study focuses on improving the design for disassembly of key components within product maintenance and recycling procedures. Modeling the product’s ease of disassembly structure is a crucial step towards achieving this goal. This section reviews the literature on product modeling and improvements in design for disassembly.

Before planning the product disassembly sequence, assessing ease of disassembly, and optimizing designs, a systematic information model is essential for representing product information (Luo, Peng, 2016)^14^. Common modeling approaches include graph-based modeling, Petri network (PN) modeling, matrix-based modeling, and CAD-based path planning^15^. Among these, graph-based methods, such as with/or diagrams and disassembly prioritization diagrams, are the earliest and most prevalent models for product disassembly. Matrix-based models, which include contact matrices for fasteners, translation matrices for components, and interference matrices for disassembly priorities, are also widely used^16^. Most research papers favor graph-based representations in disassembly sequence studies because they effectively reveal all feasible disassembly sequences and provide a reliable basis for optimal sequence planning^16^. However, these models provide constraints on components, generally used for calculating disassembly sequence optimization, but lack the disassembly information needed for easy-to-disassemble design optimization.

Paul Vanegas (2018) et al. devised the “eDiM” (ease of Disassembly Metric) technique to enhance product disassembly design^17^. This technique measures how much each task contributes to the total disassembly time, thereby identifying critical aspects for improving disassemblability. However, the optimization strategy primarily focuses on converting long-duration tasks into shorter ones, which may overlook product reliability in practice. Moreover, this approach only provides disassembly information without reflecting the priority constraints of components. To address this, recent studies have introduced visual and colorful disassembly diagram models. For designers, visual representations of the disassembly process are powerful tools in making design decisions^18^. Giovanni Formentini (2023)^19^ introduced the Parent-Action-Child (PAC) model for cyclic disassembly design. This model enhances the modeling and visualization of product disassembly information by considering different disassembly scenarios and providing more detailed steps for complex products. Francesco De Fazio(2021)^20^ proposed an innovative approach to visualize product disassembly diagrams, incorporating new standard visual elements and attributes to highlight key components and design parameters, thereby guiding product redesign. The model combines multiple parts together by clustering to reduce the disassembly steps required to reach the target part. However, its disassembly sequence is artificially set and lacks the flexibility to change for different disassembly targets.

These methods can optimize components with high disassembly difficulty effectively. However, when dealing with products that have numerous parts and complex structures, the multitude of modeling steps leads to difficulties in modeling. These methods fail to comprehensively consider the distribution of components within the product structure, which may result in suboptimal or less effective optimization strategies. To overcome this limitation, this paper proposes a visual model that not only provides disassembly information but also systematically analyzes the hierarchical depth and interdependencies of components within the overall product structure, ensuring that the proposed optimization strategies are more efficient and practical.

##  Methods

**Fig. 1:**
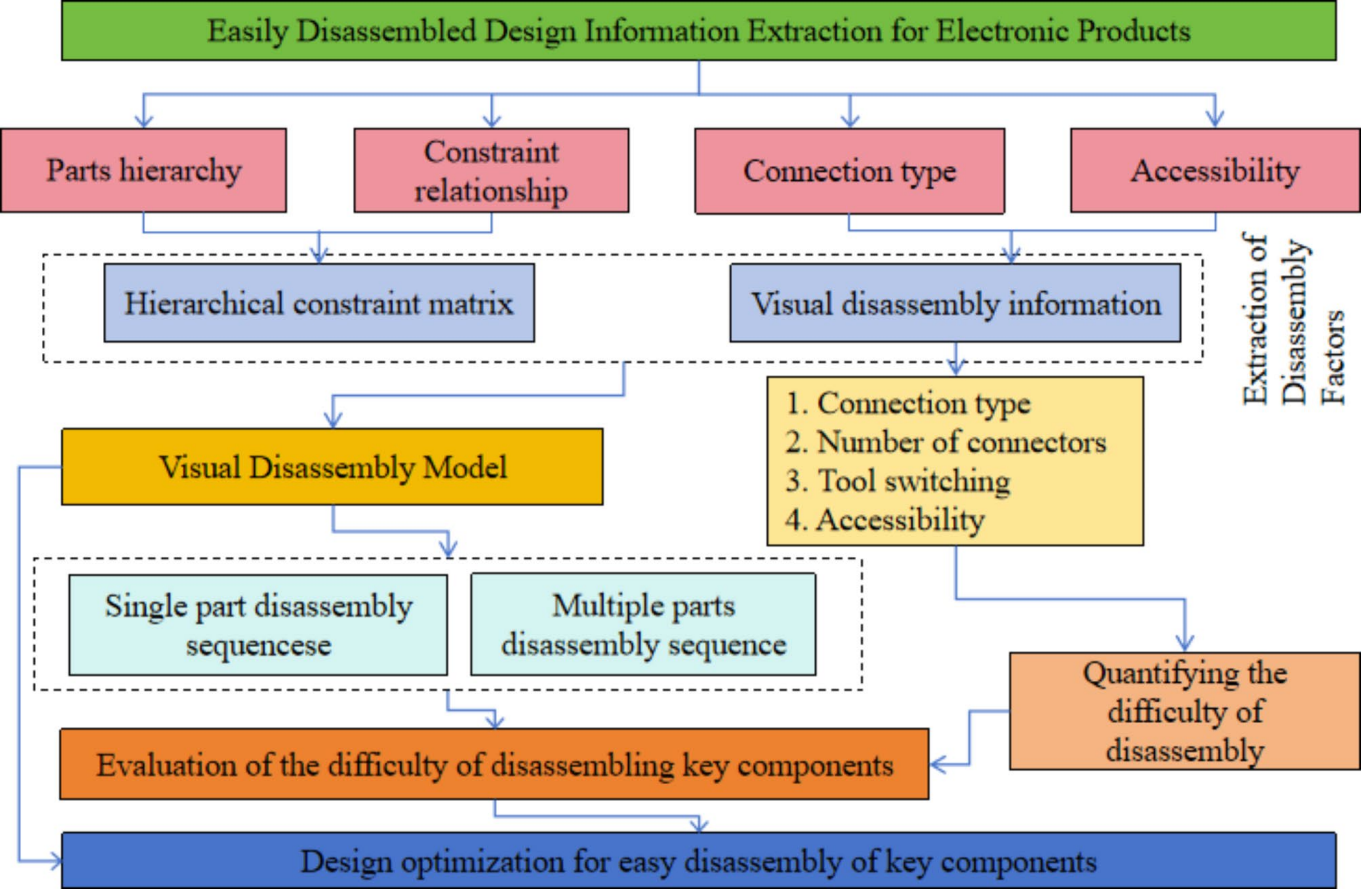
Optimization method for easy disassembly design.

This study introduces a novel visual modeling approach termed the Peony Diagram (see Fig. 1), designed for early-stage product development to facilitate the disassembly of key components under various recycling scenarios. The model offers designers insights into the product’s overall structure, disassembly levels, and component disassembly specifics. By integrating the MOST (Maynard Operation Sequence Technique) method and the Design for Disassembly Index (DEI) scorecard, the model quantifies disassembly information. This enables the evaluation of product disassembly difficulty across different strategies and serves as a foundation for optimizing the design of easily disassembled key components.

###  Peony diagram modeling

Product modeling forms the foundation for assessing disassembly difficulty, with the accurate construction of a disassembly information model being pivotal in capturing the priority constraint relationships among product parts. This model must precisely depict the hierarchical structure and disassembly priority of each component to establish a sequence for disassembling target parts. Moreover, the model’s ability to visually represent disassembly information offers a basis for optimizing designs that facilitate easy disassembly.

#### Part hierarchy diagram modeling

**Fig. 2:**
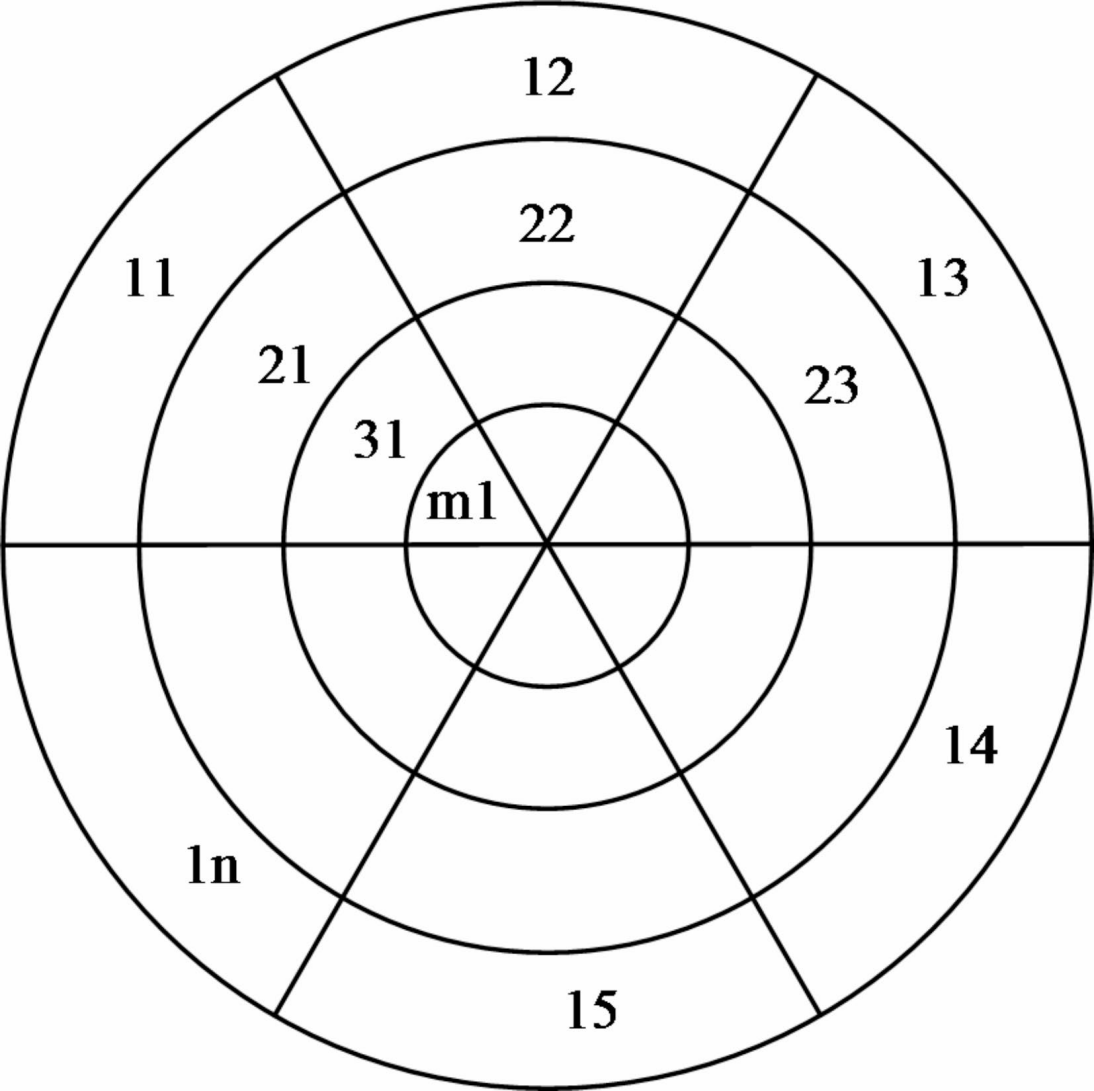
Peony diagram model.

As depicted in Fig. 2, the Peony Diagram model is structured as an *m×n* torus, comprising m concentric circles representing different layers of part placement. The outermost to innermost circles correspond to the 1st through mth layers respectively, each divided into an average of n sequences. In the model, parts are assigned part numbers, and these are placed within their respective sequences. Within each layer, sequences are juxtaposed, and each sequence imposes constraints upwards on sequences in higher layers but not on those in the same sequence in lower layers. For instance, parts in sequence 11 constrain part 21, as well as 31 through m1, but not parts in sequences 22 through 2n.

To derive the disassembly sequence of key parts, the hierarchical constraint matrix is initially constructed. This matrix primarily delineates the priority constraint relationships among parts. In the hierarchical constraint matrix, rows represent the hierarchical tiers of the Peony Diagram, while columns denote the sequences within each tier. The modeling process involves the following steps:

1. Determining Part Hierarchy: Each of the matrix’s 1st through *m*th rows corresponds to the 1st through *m*th hierarchical tiers in the Peony Diagram. Parts in a current tier can be disassembled after removing all upper-tier parts, without affecting other parts. This determination focuses solely on part hierarchy, disregarding connection methods, dismantling procedures, and steps.
2. Establishing Inter-tier Part Constraints: Parts that constrain others occupy the same column. If part P1 constrains multiple parts, it is represented in the respective sequences of the tier above each constrained part. Similarly, if part P2 is constrained by multiple parts, it is indicated in the corresponding sequences of the tier below each constrained part.
3. Transforming Hierarchical Priority Matrix into Peony Diagram: An *m × n* hierarchical priority matrix corresponds to an *m × n* toroidal diagram in the Peony model. Here, each part’s serial number is placed in its designated sequence position, with distinct colors used to differentiate each part.

A special scenario in disassembly planning involves parts groups: for complex assemblies where removing a part necessitates dismantling others in sequence, these interconnected parts form a cohesive unit termed a parts group. In the Peony Diagram, such groups are represented by a single serial number, simplifying the representation of interconnected disassembly dependencies.

**Fig. 3:**
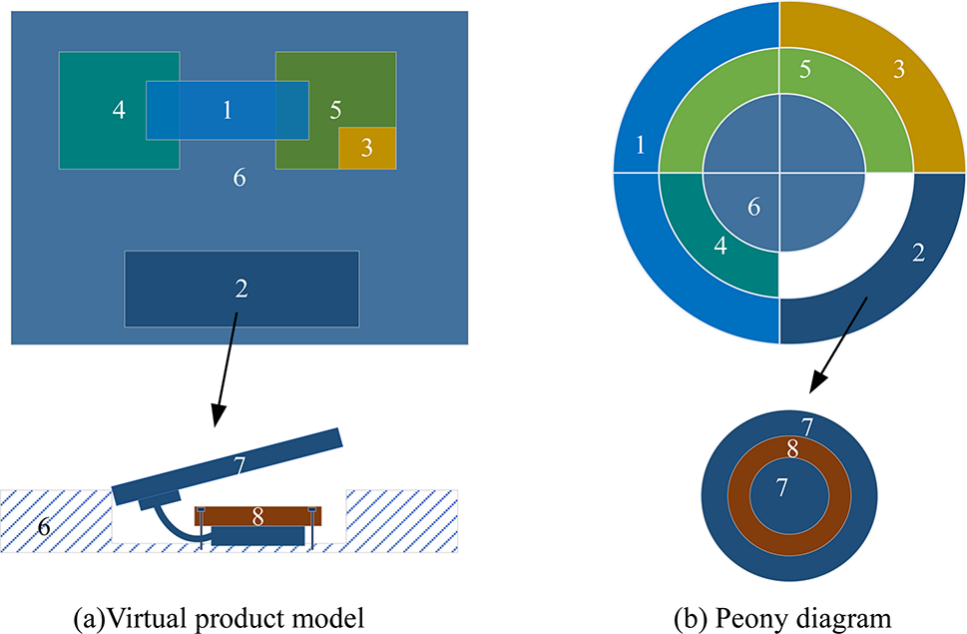
Case modeling.

A virtual product model is illustrated in Fig. 3(a), depicting a top view. The model consists of six parts, of which parts 4 and 5 and part group 2 (with sub-parts 7 and 8) are fixed to part 6. Part 3 is mounted on part 5, while part 1 is positioned on top of parts 4 and 5. The sectional view of part group 2 is indicated by the arrow.

Its hierarchical prioritization matrix (see Table 1) is established through the following steps: (1) Determining Parts Hierarchy: Layer 1 includes parts 1 and 3, as well as part group 2; Layer 2 comprises parts 4 and 5; and Layer 3 consists of part 6. (2) Establishing Par t Constraint Relationships: Part 5 is constrained by both parts 1 and (3) In the hierarchical matrix (Table 1), parts 1 and 3 are positioned in the same sequence one level above part 5, this indicates that part 5 occupies two sequences rather than the existence of two part 5. Similarly, part 1 constrains both parts 4 and 5. Thus, in the hierarchical matrix (Table 1), parts 4 and 5 are placed sequentially at the same level, reflecting part 1’s constraints across these components. The hierarchical prioritization matrix is transformed into a Peony Diagram, illustrated in Fig. 3(b).

**Table 1:** Hierarchical constraint matrix.

1	1	2	3
4	5	0	5
6	6	6	6

Specifically, parts group 2 comprises a cover plate(7) and a fixing piece (8). The fixing piece (8) secures the row of wires of the cover (7) with screws. The process of removing part 2 involves opening the cover (7), detaching the fixing piece (8), removing the wires, and then finally removing the part itself. Parts 7 and 8 together form a unit referred to as part group 2. In the overall modeling, part group 2 is represented as a three-layered sub-diagram within the Peony Diagram, as depicted in Fig. 3(b).

The Peony diagram serves as the foundation for planning the sequence of parts in subsequent evaluations of disassembly difficulty, facilitating the intuitive determination of the shortest disassembly paths for both single and multiple target parts. The shortest path planning method is outlined as follows:

1. Determining the Shortest Path for Individual Components: Begin from the sequence level where the part to be disassembled is located. Query all sequences from the previous level that constrain the part, repeating this process until reaching the topmost level. If a sequence in the previous level is empty and does not constrain any part, continue querying the previous level until reaching the topmost level.
2. Determining the Shortest Path for Multiple Components: Similarly, start with the highest hierarchical target part and sequentially query upper hierarchical constraints and target parts up to the topmost hierarchical level. This method is detailed further, including specific cases and validation, in section “Case study”.

This approach ensures systematic planning of disassembly sequences based on the Peony diagram’s hierarchical structure, enhancing clarity and coherence in disassembly path determination.

#### Disassembly infographic

While the part hierarchy diagram can visually depict the shortest disassembly paths for individual parts or multiple parts disassembled simultaneously, it lacks the ability to convey disassembly difficulty information. To address this, disassembly icon symbols are incorporated into the model. These symbols represent detailed disassembly process information, enhancing visualization capabilities within the model.

1. Connection type icons

The type and quantity of connections among parts are critical factors that directly influence disassembly difficulty. In the model, icons representing connection types and quantities are assigned to each part. These icons serve as a foundation for subsequent assessment and analysis of disassembly difficulty. Table 2 illustrates graphical symbols for connection types commonly found in mobile electronics.

**Table 2:** Icons of connection types.

Connection type	Icons	Description
Y-screw		If a part is fixed by a Y-shaped screw, the connection form of the part is indicated by the symbol.
Cross screws		If the part is secured by Cross-shaped screws, the form of connection of the part is indicated by this symbol.
Pentagonal screws		If the part is secured by a pentagonal screw, the form of connection of the part is indicated by this symbol.
BTB Male		The end of the part with the row of wires is the male end, remove the BTB to get the part, then the connection form of the part is indicated by this symbol.
BTB Female		The other end of the part that is plugged in by the male end becomes the female end.
Adhesive		If a part is connected by means of adhesive, and the part can be removed by removing the adhesive, the form of connection of the part is indicated by this symbol.
Snap		If a part is connected by means of a snap and can be removed by opening the snap, the form of connection of the part is indicated by this symbol.
RF coaxial connection		If the part is connected by a coaxial cable, the form of connection of the part is indicated by the symbol

2. Accessibility icons

During the actual disassembly of a product, factors beyond connection type and quantity can significantly impact disassembly difficulty. These include tool switching, accessibility of connections, potential part damage, and alterations in material properties. Tool switching is often influenced by the type of part connections. However, since this study focuses on evaluating product disassembly ease during the design phase, discussions on part damage and material property changes are omitted. Table 3 outlines the accessibility of connections for reference. where the icons and descriptions refer to Table 6 in Francesco De Fazio’s paper, with icon extensions.

**Table 3:** Icons of accessibility^20^.

Type	Icons	Description
Invisible		Hidden connectors, difficult to reach or identify with tools
X/Y-axis		The screws are on the side, you need one hand to fix the phone, screwdriver to screw the screws from the side
Limited operating space		Operating pitch less than 0.1 mm
Backside		Connectors are not on the same side as the part and need to be removed from the back side

3. Key component icons

This study centers on the disassembly of critical parts, encompassing components that are High potential failure rate, Economic value, Embodied environmental impact and are recyclable. These critical parts are distinguished in the model using established graphical symbols that depict their attributes, including value, environmental hazards, fragility, and recycling potential, as shown in Table 4. The icons in the table are borrowed from Table 7 in Francesco De Fazio’s paper and have been simplified and expanded.

**Table 4:** Icons of key components^20^.

Targeted part categories	Icons	Description
High potential failure rate		This symbol indicates a part that is prone to damage or has a high failure rate.
Environmental impact		Indicates parts that are very harmful to the environment
Economic value		Indicates the component with the highest implied value
Recyclable		Indicates some functional recycling after disassembly

### Ease of disassembly assessment

**Fig. 4:**
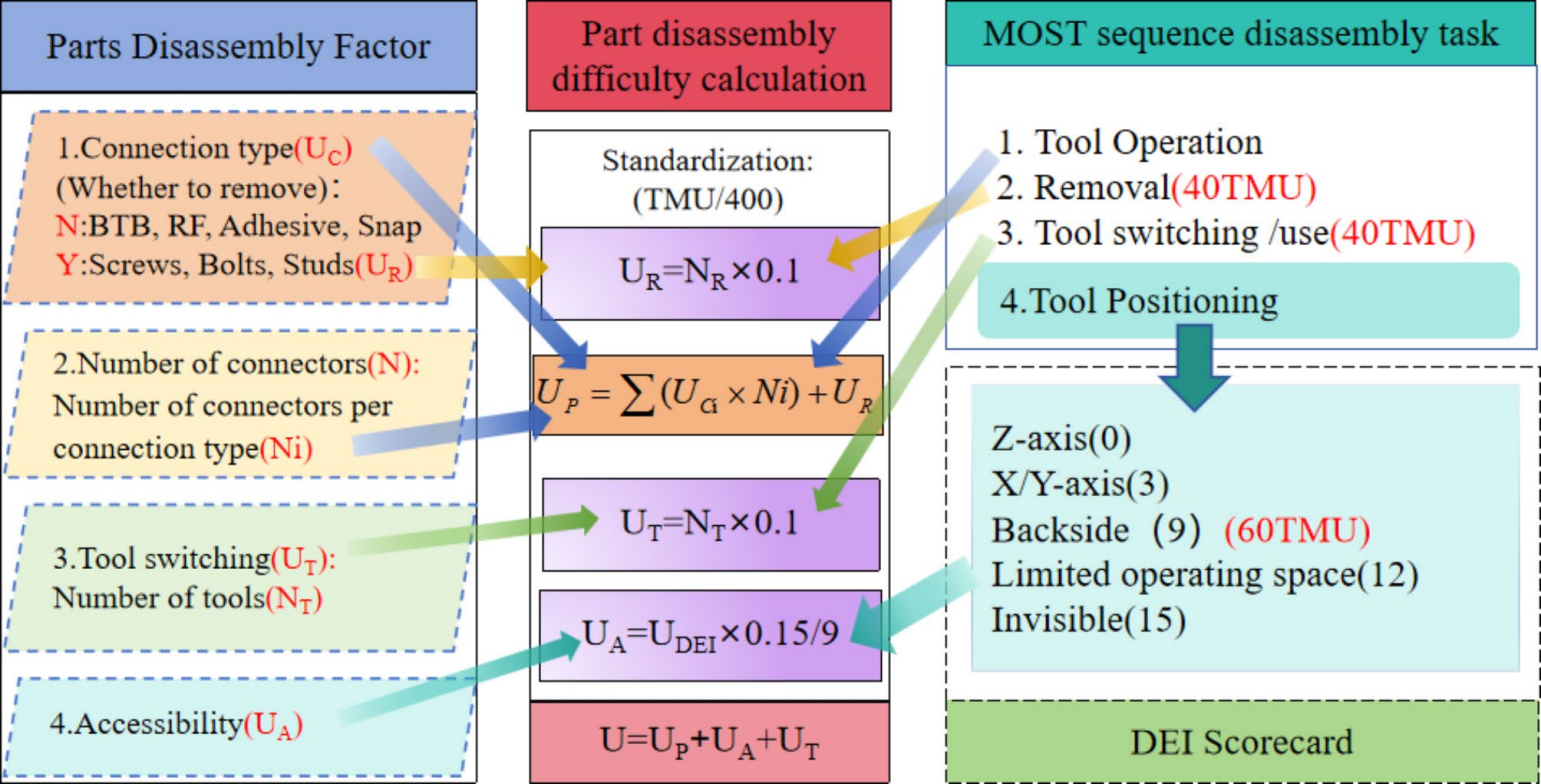
Quantifying the difficulty of disassembling parts.

The Peony diagram model can visualize the disassembly hierarchy and disassembly information of the parts. In order to provide designers with the evaluation value of component disassembly difficulty, this paper adopts the easy disassembly metric (eDiM), which is a quantitative assessment method for evaluating disassembly workload^21^. This method is an “eDiM” (ease of Disassembly Metric) method that calculates disassembly time based on the Maynard Operating Sequence Technique^22^. In this study, we analyzed the disassembly information and extracted four part disassembly factors related to the design, including tool switching, connection type, number of connections, and accessibility information. To quantify these factors and calculate the difficulty of part disassembly, we utilize four tasks from the MOST sequence model: tool switching/use, tool positioning, tool manipulation, and removal. By examining the relationship between these four disassembly factors and the corresponding MOST tasks, we apply the MOST sequence times to quantify the factors. Moreover, to accurately quantify the accessibility factor, this study employs a hybrid approach using both MOST and DEI (Disassembly Effort Index) scorecards, as depicted in Fig. 4.

The Maynard Operation Sequence Technique (MOST) serves as a standardized measure for operational time. As a modeling approach, MOST breaks down actions into fundamental operations (Zandin, 2002)^23^. This technique is founded on the identification of three primary sequences of actions: general actions, control actions, and tool usage. Each sequence is assigned a time coefficient, which is then translated into a time value. In this study, the time required for Maynard’s operational tasks is utilized as a benchmark to calculate disassembly difficulty values based on disassembly information. Table 5 presents the Maynard standard action sequence, which guides the time evaluation of each task in the disassembly process.

**Table 5:** MOST sequence model used to describe the operation of unfastening and removing a screw^20^.

MOST sequence for screw unfastening and removal						Description
A1B0G1	A1B0P3	L10	A1B0P1	A1B0G1	A1B0P1	
Get tool	Put tool in place action	Tool action	Put tool aside	Reach and grab the screw	Remove the screw	
1.Basic actions A = Action Distance B = Body Motion G = Gain Control P = Placement						A1 = Reach to a screw driver within reach B0 = No body motion occurs in vertical direction G1 = Gaining control of the screw driver
2.Tool Use Action L = Loosen C = Cut						P1 = Places in place without pressurization P3 = Dual action with some adjustment when placing an object
3.Control Actions M = Move Controlled						L10 = Finger turn screwdriver (8 turns) to loosen threads

The sequence involves picking up a screwdriver from a nearby location, approaching the screw, positioning the screwdriver on the screw, rotating the screwdriver eight times manually to loosen the screw, grasping the screw with one hand, and returning the screwdriver to its initial position. These operations are broken down into the disassembly tasks outlined in Table 6.

**Table 6:** Determination of dismantling tasks and difficulty coefficient^24^.

Disassembly task	Description	Sequence	TMU
Tool switching/use	Placement and Grabbing Tools	A1B0G1 + A1B0P1	40
Tool Positioning	Position the screwdriver on the screw by hand	A1B0P3	40
Tool Operation	Start rotating to loosen the screw	L10	100
Removal	Remove and place screws	A1B0G1 + A1B0P1	40

The MOST sequence time for each disassembly task can be seen in Table 6, which is calculated by adding the serial numbers in the sequence of operations and multiplying by 10 in TMU (Time Measurement Unit)^24^. The disassembly factors are quantified by examining the correlation between disassembly tasks and factors, utilizing the MOST time for these tasks, as depicted in Fig. 4. Specifically: (1) **Tool Switching/Usage and Tool Manipulation**: The disassembly difficulty for these tasks is primarily influenced by the type and quantity of part connections. Consequently, the difficulty associated with the type of part connections can be measured using the MOST time for tool manipulation. Meanwhile, the disassembly difficulty related to the quantity of connection types can be assessed by multiplying the MOST time for tool switching by the number of connection types. (2) **Tool Positioning**: This task’s difficulty is dictated by the accessibility of the connections, and it is quantified by the MOST time for tool positioning. (3) **Removal Tasks**: These are contingent on the type of connections. For instance, connectors like screws, bolts, and studs need to be removed, whereas adhesives, BTB connectors, RF coaxial cables, and snaps do not require removal. Therefore, when calculating the disassembly difficulty for parts that necessitate the removal of connections, the operation time for removal should be added. The standard actions for each part removal operation are consistent, so to streamline the evaluation process, the removal task can be omitted when assessing parts.

1. **Type of connections**.

The primary types of electronic component connections include gluing, threading, BTB connecting wires, RF coaxial lines, and snap fasteners. Specifically, disassembly actions for snap fasteners, BTB connecting wires, RF coaxial lines, and threaded connections can be described using the “C(1,3)” control motion parameter or “L(6,10)” tool action motion in the MOST sequence, as detailed in Table 7.

In this study, the difficulty of dismantling glued parts is categorized into three levels: difficult, medium, and easy. (1) **Difficult**: This level involves using vacuum suction cups to secure parts, forcefully inserting opening picks into adhesive areas, and cutting through the adhesive. For instance, to remove a smartphone screen, the standard action involves pulling the suction cup away from the screen and inserting opening picks into the screen gap along each side, making 4 cuts. (2) **Medium**: Medium difficulty disassembly requires the use of a pry bar to separate glued parts. (3) **Easy**: Parts at this level can be easily separated by hand. The sequence and detailed descriptions for each level are provided in Table 7.

**Table 7:** Disassembly difficulty coefficient of connection type.

Connection type	Description	Sequences	TMU	Difficulty coefficient Ui
Screw	Threaded connections in smartphones are generally in the range of 3–8 turns	L6-L10	80	0.2
BTB	Use a crowbar to pry open the BTB	C1	10	0.025
RF	Use a crowbar to pry open the RF	C1	10	0.025
Clips	Use a crowbar to open the clips	C1	10	0.025
Adhesive	Difficulty: It requires a lot of force to insert the tool into the glued part and then it is difficult to remove it.	M8 + C16 + 16	400	1
	Medium: needs to be removed with a little force using a pry bar	C6-C10	80	0.2
	Easy: can be easily removed by hand	M1	10	0.025

To facilitate subsequent calculations of disassembly difficulty, the MOST times in Table 7 were normalized by dividing each MOST time by 400 to obtain the difficulty coefficient U_C_ for the connection type.

(2) **Tool switching**.

Parts disassembly can be done manually and with tools; manual disassembly eliminates tool use and tool change time; tool use and switching MOST disassembly time are both 40 TMU. therefore, tool switching MOST disassembly time is calculated as:

1 $${{\text{T}}_{{\text{tool}}}}={\text{Nt}} \times {\text{4}}0{\text{TMU}}$$

*Nt* is the number of tool switches in part disassembly, which is related to the number of connection types in the part.

(3) **Accessibility**.

Reachability refers to the difficulty in locating and approaching connectors, influenced by factors such as direction, visibility, and accessibility of connectors. The MOST method provides limited guidance on this aspect, offering only partial references. In this paper, reachability disassembly difficulty is quantified using difficulty values derived from the Design for Disassembly Index (DEI) scorecard proposed by Das from New Jersey Institute of Technology^19^, as detailed in Table 8. The reachability difficulty coefficients are determined by integrating DEI scorecard^25^assessments with standard positioning maneuvers from the MOST method.

**Table 8:** Disassembly difficulty coefficient of accessibility.

Type	MOST Description	DEI Description	TMU	DEI	Difficulty coefficient U_A_
Z-axis	Vertical direction	Directly above the product	10	0	0.025
X/Y-axis	Screws on the side	From the side of the part	30	3	0.067
> 6”deep	—	Greater than 6 inches deep	—	6	0.108
From below	Insert the plug into the socket under the table or add nuts to the covered bolts	A negative Z-axis access	60	9	0.15
Limited operating Space	—	when the disassembly point is accessed through a flexible or bent tool	—	12	0.192
Invisible	—	When the disassembly is inside the product and not visible	—	15	0.233

This study employs the DEI (Difficulty of Accessibility Index) to quantify the accessibility challenge in disassembly. The DEI scoring system reflects how easily a tool can access a connector based on various localization methods. While the MOST (Maynard Operation Sequence Technique) sequence includes localization methods, they are not exhaustive. To align the DEI scores with the quantitative results of other disassembly factors, we convert DEI scores to MOST time by establishing a relationship between the two, thus calculating the accessibility difficulty factor.

As shown in Table 8, there are only three samples of MOST time data. Given the limited amount of data, this study decided to use the Endpoint Method to determine the linear relationship between DEI scores and MOST time data. The formula for fitting a straight line is as follows:

2 $${{\text{T}}_{{\text{MOST}}}}={\text{1}}0+({\text{6}}0 - {\text{1}}0) \times {{\text{S}}_{{\text{DEI}}}}/{\text{9}}$$

where T_MOST_ is the MOST time and S_DEI_ is the DEI score.

The missing reachability MOST time is calculated using this formula and the reachability difficulty factor is obtained by normalizing: U_A_ = T_MOST_/400, and the quantitative results are detailed in Table 8.

(4) **Total disassembly difficulty calculation**.

The calculation for disassembly difficulty based on the number of connectors in a part, which may have various connection types with multiple connectors each, involves differentiating between connectors that need to be removed and those that do not. Hence, the disassembly difficulty for part connectors is computed as follows:

3 $${{\text{U}}_{\text{P}}}=+{{\text{U}}_{\text{R}}}$$

Where *i* is the type of connection type, *U*_*Ci*_ is the difficulty coefficient of the *i*th connection type, *Ni* is the number of connectors of the *i*th connection type, *U*_*R*_ is the difficulty of removing the connectors of the part, *U*_*R*_*=0.1×N*_*R*_, and *N*_*R*_ is the number of connectors that need to be removed from the part.

In this study, the eDiM method was used, on the basis of which the disassembly factor MOST time was normalized and the disassembly difficulty of each assessment factor was calculated. Therefore, the total disassembly difficulty is the cumulative of these disassembly difficulties.

4 $${\text{U}}\,=\,{{\text{U}}_{\text{P}}}+{{\text{U}}_{\text{A}}}+{{\text{U}}_{\text{T}}}$$

*U*_*P*_ is the connection disassembly difficulty of the part, *U*_*A*_ is the accessibility difficulty of the part, and *U*_*T*_ is the tool switching difficulty of the part.

It is hereby clarified that the part disassembly difficulty calculations herein are intended to provide designers with an assessment of disassembly difficulty, and that only the four key factors related to the design process that have a direct impact on disassembly have been studied and calculated, and that other factors in actual disassembly have not been fully considered. Therefore, the actual disassembly time cannot be calculated using the time conversion in the MOST method.

## Case study

###  Object selection

The smartphone is chosen as the case object for this study because it encompasses a wide array of components and connection types, showcasing the structural intricacy characteristic of electronic products. Additionally, it incorporates diverse fasteners and complex component relationships. Moreover, significant structural differences among brands make smartphones ideal for comparative analysis, thereby enhancing the research’s credibility. The methodology of this study is also applicable to other small electronic products, such as computers and tablets, which our research team has modeled and validated. Additionally, for small household appliances like hair dryers and electric kettles, this method has shown good applicability in modeling, although disassembly information still needs further supplementation. Modeling for large equipment remains to be explored.

###  Experimental methods

To model the disassembly information of a product, it is essential to first gather all relevant data needed for the disassembly process. In this study, manual disassembly was used, and each phone was disassembled and reassembled three times, with the procedures documented through both top-view and side-view video recordings. During the disassembly operation, two aspects need to be recorded in detail in this study:

1. The prioritization constraint relationship of the parts and the establishment of the part hierarchy constraint matrix in accordance with the hierarchical disassembly order. The current tier part is determined by identifying the part as being in the current tier if no other parts need to be removed when the part is disassembled. The constraint relationship is established by iteratively installing and disassembling all currently disassembled parts to evaluate the part’s relationship to the current part constraint. The tier constraint matrix is recorded and progressively refined throughout the disassembly process.
2. Part disassembly information, recording the connection type, number of connections, accessibility, and number of tool switches for each individual part, and organizing this data into a disassembly information spreadsheet.

The Peony diagram model is constructed by using the hierarchical constraint matrix and the disassembly information spreadsheet in order to visualize the easy-to-disassemble design. With the help of this model the shortest disassembly paths of components can be quickly identified. The disassembly difficulty of each component is calculated based on the evaluation method described in section “Ease of disassembly assessment”. Through the disassembly difficulty of each component in the shortest disassembly path, the disassembly difficulty of critical components is calculated. Finally, the easy disassembly design is optimized by combining the disassembly information in the Peony Diagram model and the level where the parts are located.

### Results

#### Modeling of peony diagram

In this case, the “Smartphones A” and “Smartphones B”, which were released at the same time, were selected as the objects for disassembly. By applying the theory introduced in section “Methods” to model and evaluate these two smartphones, and combining the key component disassembly paths and disassembly time data from the official iFixit website for the corresponding phone models, the effectiveness and accuracy of this method were verified.

1. Data Collection and Modeling.

Table 9 details the disassembly information for the “Smartphones A”, documenting the type and number of connections, accessibility, and tool switching for each part during disassembly. Connections, such as screws, bayonets, and BTB (Board-to-Board) wiring, are considered part of the component they directly attach to and are not listed separately. In the “Smartphones A”, there are two BTB connection cables, each equipped with a BTB male connector at both ends. These cables span across the battery, connecting the motherboard and the tailboard, and can be removed individually, not being specific to any single part. The assignment of BTB connectors is based on the minimum number of BTBs that must be removed during the actual disassembly of the part. For the “Smartphones A”, two BTBs are assigned to the battery, one to the secondary board, and one to the screen. Notably, the screen can be detached without removing the tail plate. However, the disassembly process for the screen is challenging due to the BTB wiring, which must be maneuvered through a narrow opening.

**Table 9:** “Smartphones A” disassembly information.

No.	Part	Invisible	Cross screws	BTB Male	BTB female	RF	Number of Tool Changes	Accessibility
1	Screen	D(Difficulty)		2			3	I(Invisible) B(backside)
2	Battery	E(Easy)		3			1	
3	Motherboard		1		All	3	2	
4	Camera modules	E		3			1	
5	Forward camera			1			1	
6	Linear Actuator Vibration Motors						0	
7	Motherboard cover and Charging Coil		5				1	
8	Lens Protection Kit and Flash		3	1			2	
9	Daughterboard cover		4				1	
10	Back cover	D					2	I
11	Daughterboard			2			1	L (Limited operating Space)
12	Speaker		3				1	
13	Receiver	E					0	
14	Metal cover		2				1	
15	Frame						0	
Total		2D + 3E	18	12		3	17	

Table 10 provides the disassembly information for the “Smartphones B”, where the screen assembly is designated as the “Screen Parts Group.” The disassembly of the iPhone 12’s screen is intricate. Unlike other devices, the screen cannot be removed immediately upon opening. To detach the screen, it is necessary to first remove two metal cover plates that constrain its BTB wiring. Only after prying open the BTB connections can the screen be fully detached. The “Screen parts Group” encompasses the screen and the two metal covers, with the screen identified as the key component.

**Table 10:** “Smartphones B” disassembly information.

No.	Part	Invisible	Y- screw	Cross screws	Phillips stud	Pentagonal screws	BTBMale	BTB female	Number of Tool Changes	Accessibility
1	Screen	D	6			2Y	3		5	I
2	Battery	D					1		2	L
3	Mainboard			3	1			All	3	
4	Back camera						2		1	
5	Face ID		1				3		1	
6	Vibration motor			1	2		1		2	
7	Back camera cover		5						1	
8	Flash light	E	2				1		2	
9	Tailgate cover		1		2				2	
10	Back cover									
11	Tail plugs	M (Medium)	3Y2Z	2X	4		2		5	3Y + 2X
12	Speaker			4					1	
13	Charging coil	M		6X			1		3	6X
14	Card slot cover 1		2						1	
15	Card slot cover 2		3						1	
16	SIM Card slot				1		1		2	
17	Antenna components	E	2	2:X + Y			1		3	
18	Fixed plastic pad		1		2				2	X + Y
Total		2D2M 2E	28	18	12	2	16		37	

Based on the modeling method described in section “Peony diagram modeling”, hierarchical constraint matrices for the parts disassembly priority relationships of the two smartphones have been created. These matrices are presented separately in Tables 11 and 12.

**Table 11:** Hierarchical constraint matrices for *“Smartphones A”*.

10	10	10	10	10	10	10
14	14	14	8	8	8	8
7	7	7	7	7	7	7
5	3	3	9	12	2	1
0	13	4	11	11	0	0
15	15	15	6	15	15	15

**Table 12:** Hierarchical constraint matrices for “Smartphones B”.

1	1	1	1	1	1	1	1	1
7	7	5	17	2	14	14	15	12
4	18	0	0	0	16	16	16	0
0	8	8	0	0	3	3	6	6
0	0	0	0	13	13	0	9	0
10	10	10	10	10	10	11	11	10

This study focuses on maintenance and recycling disassembly, with an emphasis on analyzing the disassembly difficulty of key components. Key components are those that are prone to damage, have a high environmental impact, are of high value, or are significant for recycling. According to the literature, the key components in smartphones, which are highlighted in Table 13, include these critical parts^26–28^.

**Table 13:** Key components for smartphones.

Part name	High potential failure rate	Embodied environmental impact	Economic value	Remanufacture or reuse
Screen(Blue)	√	√		√
Battery(Red)	√	√		√
Mainboard(Gold)		√	√	√
Camera(Green)				√

**Fig. 5:**
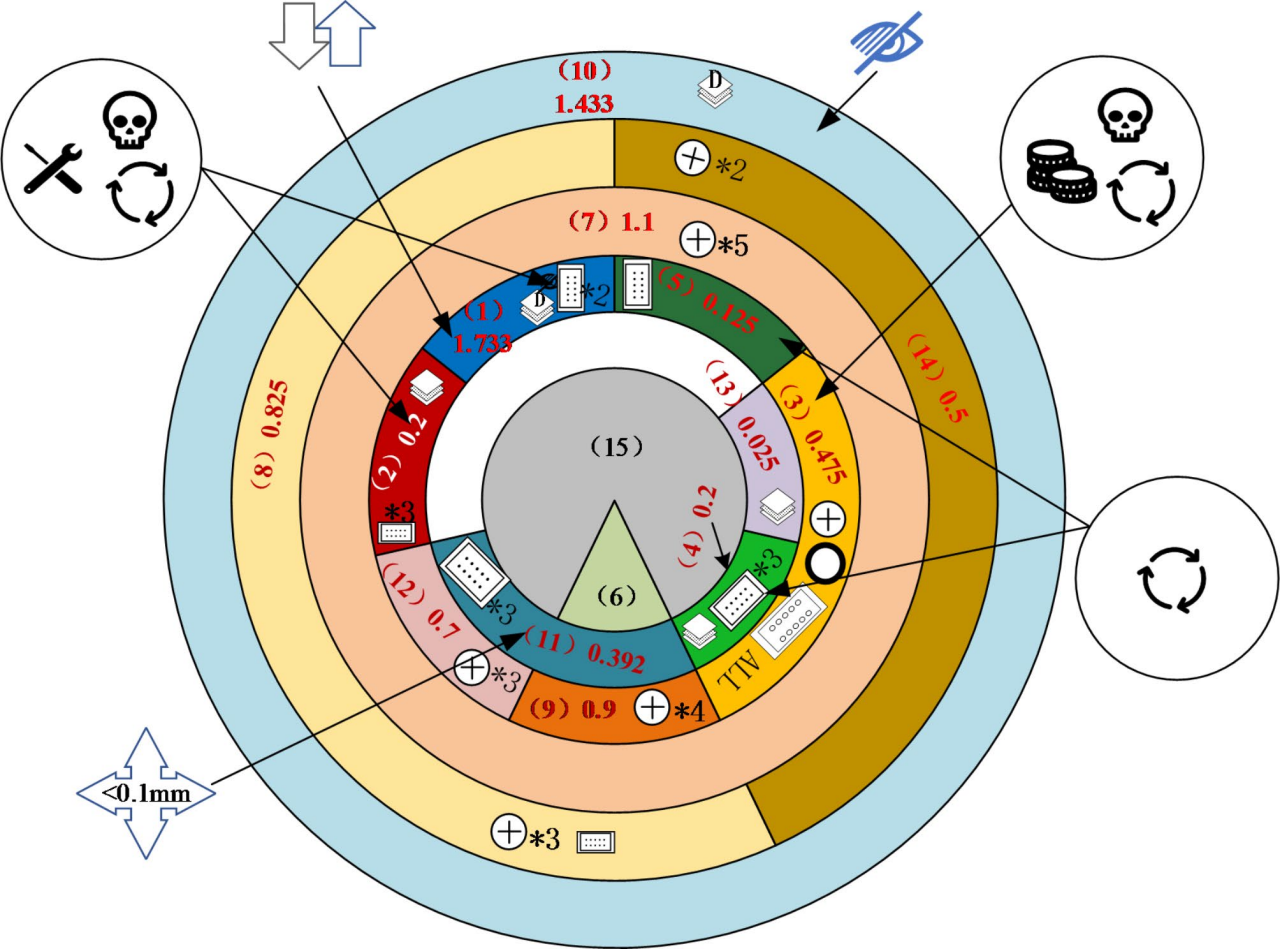
“Smartphones A” Peony Diagram.

**Fig. 6:**
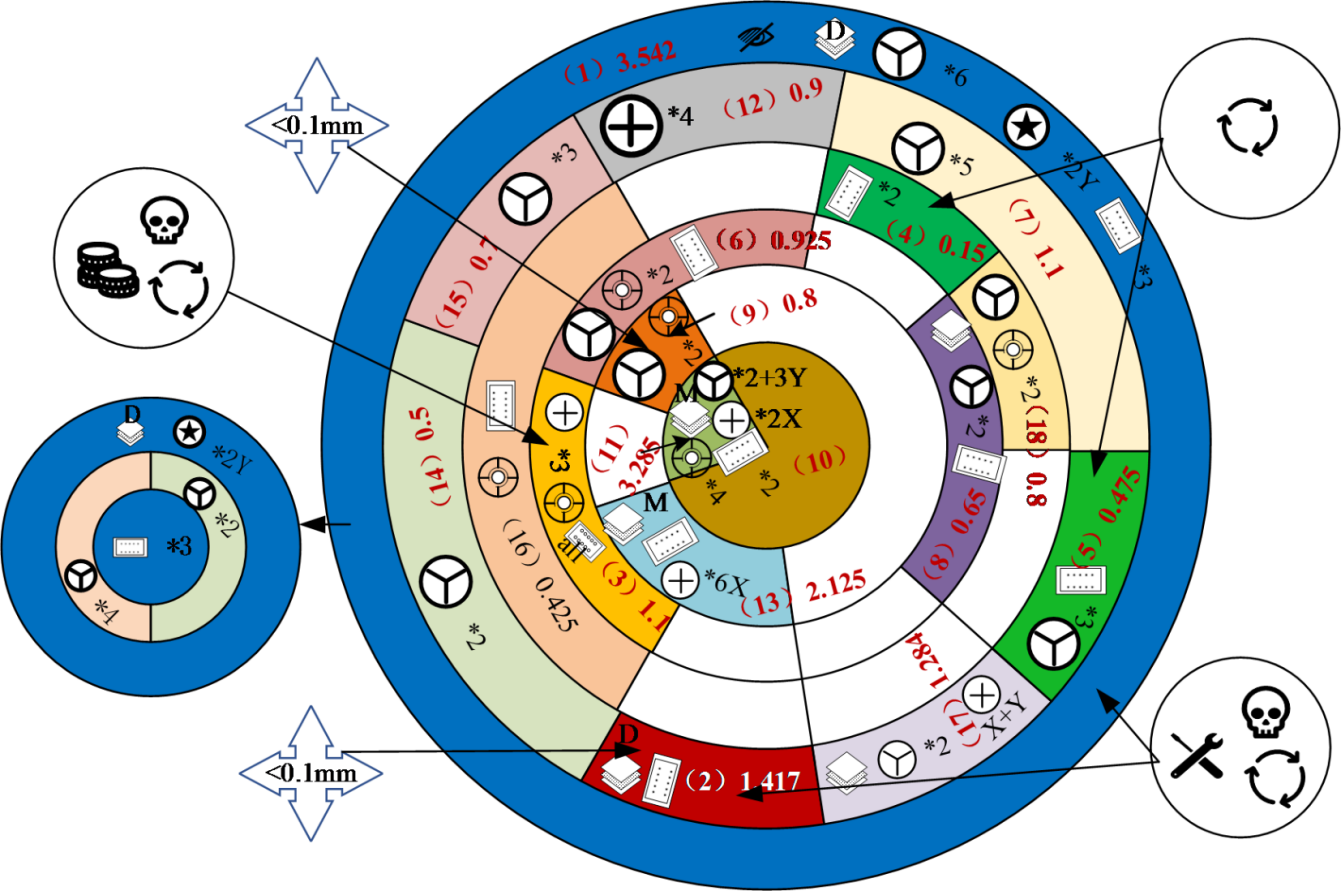
“Smartphones B” Peony Diagram.

Based on the hierarchical prioritization matrix of component disassembly and the information from each disassembly indicator, Peony diagrams for their disassembly processes are presented. These diagrams are illustrated in Figs. 5 and 6.

(2) Shortest disassembly sequence generation and validation.

The Peony diagram serves as a foundation for subsequent evaluation and optimization of disassembly difficulty. It provides essential information for disassembly, including the distribution of each component level, constraint relationships, types and quantities of component connections, and accessibility. From these diagrams, we can intuitively determine the shortest disassembly path for each component and plan the shortest disassembly path for multiple target parts.

As depicted in Fig. 5, the critical component disassembly paths for the “Smartphones A” are as follows: the shortest sequence for Screen 1 is 10-14-8-7-1 or 10-8-14-7-1, and for Battery 2 it is 10-14-8-7-2 or 10-8-14-7-2.

The IFIXIT official website primarily focuses on disassembling and repairing smartphones, computers, and tablets. According to their disassembly process for repairing the “Smartphones A” screen, it involves the following sequence: Back Cover (10) - Metal Bracket (14) - Camera Cover (8) - Main Cover (7) - Screen (1). Similarly, the battery disassembly path includes: Rear Cover (10) - Metal Bracket (14) - Camera Cover (8) - Main Cover (7) - Battery (2). By comparing these, it is evident that the Peony diagram accurately reflects the disassembly sequence of key components.

As depicted in Fig. 6, the shortest disassembly sequence for the “Smartphones B” battery is Screen Assembly (1) - Battery (2). According to the IFIXIT official website, the battery disassembly process involves the following sequence: Screen Assembly (1) - Speaker (12) - SIM Reader Cover 1 (14) - SIM Reader Cover 2 (15) - SIM Reader (16) - Electric Motor (6) - Battery (2). This comparison demonstrates that the Peony diagram accurately identifies the shortest disassembly sequence for key components.

The Peony diagram not only identifies the shortest disassembly sequence for a part but also enumerates all other possible sequences. These sequences begin by first removing any parts at the same level or lower levels that lack constraint relationships. For example, the disassembly sequences for a battery might include paths like 1-12-14-15-16-6-2, 1-1-5-17-14-15-12-2, among others. While there are numerous paths to remove the battery, the shortest path remains 1–2, which requires no removal of peripheral parts.

Thus, the Peony diagram accurately determines both the disassembly sequence of parts and their shortest paths. This paper specifically discusses only the shortest disassembly sequences of key parts. Similarly, the feasibility of the shortest sequence for disassembling multiple parts can be verified.

#### Smartphone disassembly difficulty calculation and validation

The shortest disassembly paths for critical parts are determined from the Peony diagram, and the disassembly difficulty of each part is assessed. The overall disassembly difficulty of a part is then derived by summing the difficulties of all its disassembly paths.

1. Calculation of dismantling difficulty.

According to the disassembly difficulty coefficient calculation method outlined in section “Ease of disassembly assessment”, the disassembly information in Table 11 is quantified to compute the disassembly difficulty coefficient for each component, as presented in Table 14. Similarly, the disassembly difficulty coefficients for each component of the “Smartphones B” are highlighted in Table 15.

**Table 14:** Calculation of disassembly difficulty for each part of *“Smartphones A”*.

Number	Part	Difficulty of connection type and number	Accessibility difficulty	Difficulty of switching tools	Total disassembly difficulty
1	Screen	1.05	0.383	0.3	1.733
2	Battery	1	0	0.1	0.2
3	Motherboard	0.275	0	0.2	0.475
4	Camera modules	0.1	0	0.1	0.2
5	Forward camera	0.025	0	0.1	0.125
6	Linear Actuator Vibration Motors	0	0	0.0	0
7	Motherboard cover and Wireless Charging Coil	1	0	0.1	1.1
8	Lens Protection Kit and Flash	0.625	0	0.2	0.825
9	Daughterboard cover	0.8	0	0.1	0.9
10	Back cover	1	0.233	0.2	1.433
11	Daughterboard	0.1	0.192	0.1	0.392
12	Speaker	0.6	0	0.1	0.7
13	telephone receiver	0.025	0	0	0.025
14	Metal cover	0.4	0	0.1	0.5
15	Frame				0
	Total				8.608

**Table 15:** Calculation of disassembly difficulty for each part of the “Smartphones B”.

Number	Part	Difficulty of connection type and number	Accessibility difficulty	Difficulty of switching tools	Total disassembly difficulty
1	screen	2.675	0.367	0.5	3.542
2	battery	1.025	0.192	0.2	1.417
3	Mainboard	0.8	0	0.3	1.1
4	Back camera	0.05	0	0.1	0.15
5	Face ID	0.275	0	0.2	0.475
6	Vibration motor	0.625	0	0.3	0.925
7	Back camera cover	1	0	0.1	1.1
8	Flash light	0.45	0	0.2	0.65
9	Tailgate cover	0.6	0	0.2	0.8
10	Back cover	0	0	0	0
11	Tail plugs	2.45	0.335	0.5	3.285
12	speaker	0.8	0	0.1	0.9
13	Charging coil	1.425	0.4	0.3	2.125
14	Card slot cover 1	0.4	0	0.1	0.5
15	Card slot cover 2	0.6	0	0.1	0.7
16	SIM Card slot	0.225	0	0.2	0.425
17	Antenna components	0.85	0.134	0.3	1.284
18	Fixed plastic pad	0.6	0	0.2	0.8
	Total				20.178

(2) Dismantling difficulty verification.

Taking “Smartphones A” repair parts disassembly as an example:

1. Back Cover: The back cover can be directly removed with a disassembly difficulty rating of 1.433.
2. Screen: To remove the screen, the sequence involves removing the back cover (10), metal cover (14), camera cover and flash (8), motherboard cover (7), and finally the screen (1). The disassembly difficulty for screen repair is the sum of the difficulties of these parts in sequence, totaling 5.591.
3. Battery: The disassembly path for the battery includes removing the back cover (10), metal cover (14) + camera cover and flash (8), motherboard cover (7), and finally the battery (2). The repair difficulty for the battery disassembly is the sum of the difficulties of these parts in sequence, totaling 4.058.
  According to IFIXIT’s official website, these three parts’ disassembly paths align with the shortest paths indicated in their charts. The disassembly times for “Smartphones A” are approximately 15–25 min for the back cover, 45 min to 2 h for screen repair, and 45 min to 1 h for battery repair.
  Table 16 demonstrates that the disassembly difficulties calculated in this study show a positive correlation with those reported by iFixit. The disassembly difficulties for repairing parts using the Peony Diagram model are quantified as follows: the screen is 3.9 times more challenging to disassemble than the back cover and 1.38 times more challenging than the battery. IFIXIT provides a disassembly time range rather than a specific value for repairing the three parts, with the screen showing a particularly wide range. By calculating the upper and lower limits and their ratios, the screen’s disassembly time ranges approximately from 3 to 4.8 times that of the back cover and 1 to 2 times that of the battery. These ratios fall within the observed range, indicating that the method effectively reflects actual disassembly difficulties.
  **Table 16:** Comparison of disassembly difficulty and IFIXIT time.

Evaluation methods	Back cover	battery	screen
Difficulty of disassembling the Peonies for repair	1.433	4.058	5.591
IFIXIT Disassembly time	15–25 min	45 min–1 h	45 min–2 h

## Discussion

As depicted in Figs. 5 and 6, the “Smartphones A” and “Smartphones B” are both structured in a 6 × 7 and 6 × 9 matrix, respectively, indicating they share a total disassembly depth of 6 layers. Despite this similarity, the overall disassembly difficulty of the “Smartphones B” is significantly higher compared to the “Smartphones A”. Individual component disassembly difficulties for the “Smartphones B” are generally greater than those for the “Smartphones A”.This section discusses and analyzes two aspects: product disassembly for repair and disassembled for recycling of key components. It proposes an optimization plan for disassembly difficulty based on disassembly level, connection types and quantities, and tool switching.

###  Assessment and optimization of disassembly difficulty for repair

1. Analysis of results.

**Fig. 7:**
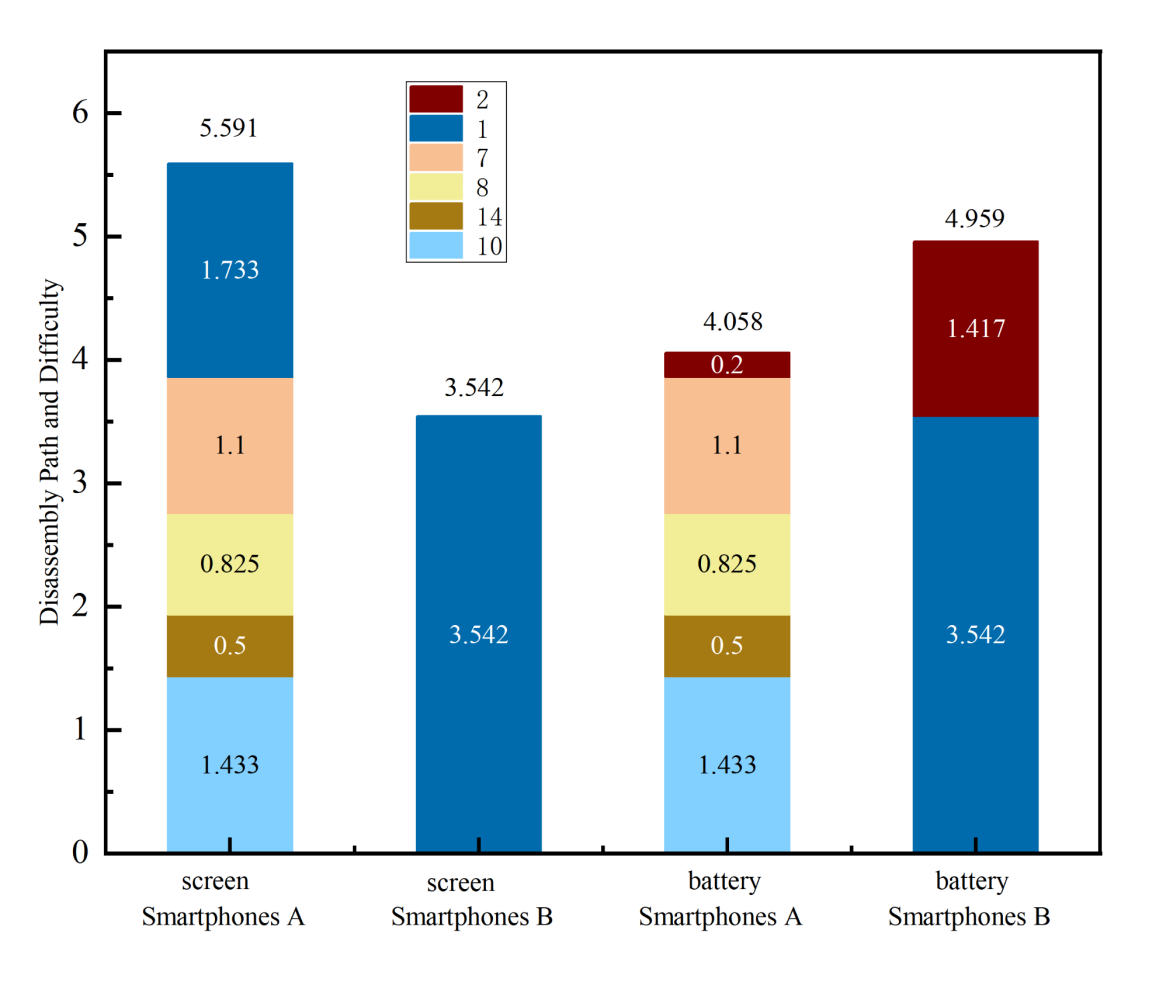
Difficulty of disassembling repair parts.

From Figs. 5 and 6, the distribution reveals the disassembly paths and difficulties for the two smartphones’ vulnerable parts, namely the screen and battery, during the repair process, as depicted in Fig. 7. The “Smartphones A” exhibits a significantly higher disassembly difficulty for the screen compared to the “Smartphones B”, while the battery repair is slightly easier than that of the “Smartphones B”. Overall, the repairability of the “Smartphones B” surpasses that of the “Smartphones A”.

According to IFIXIT’s official website, the “Smartphones A” receives a repairability score of 4 out of 10, whereas the “Smartphones B” scores 6 out of 10, indicating higher scores denote better repairability. This assessment aligns with the official website ratings, confirming that the “Smartphones B” is more repairable than the “Smartphones A”.

(2) Optimization recommendations.

The “Smartphones A” can be optimized by focusing on the disassembly level, particularly concerning the screen and battery, and addressing the disassembly difficulty of related constrained parts in its design. During the disassembly process, it was identified that the BTB interface on the screen significantly influences the disassembly level. Optimizing the BTB interface disassembly to the second layer allows for easier removal upon opening the back cover, reducing the maintenance and disassembly difficulty by 2.425, which accounts for a 43% reduction.

The “Smartphones B”’s screen and battery already belong to the first and second-tier levels, yet they involve multiple types and numbers of connectors. Therefore, optimization can focus on connector types, especially reducing the number of screws. If all 8 screws can be optimized, the disassembly difficulty would decrease by 1.9, This optimization will reduce the difficulty of screen removal to 46% of the original.

###  Assessment and optimization of disassembly difficulty for recycling

1. Analysis of results.

**Fig. 8:**
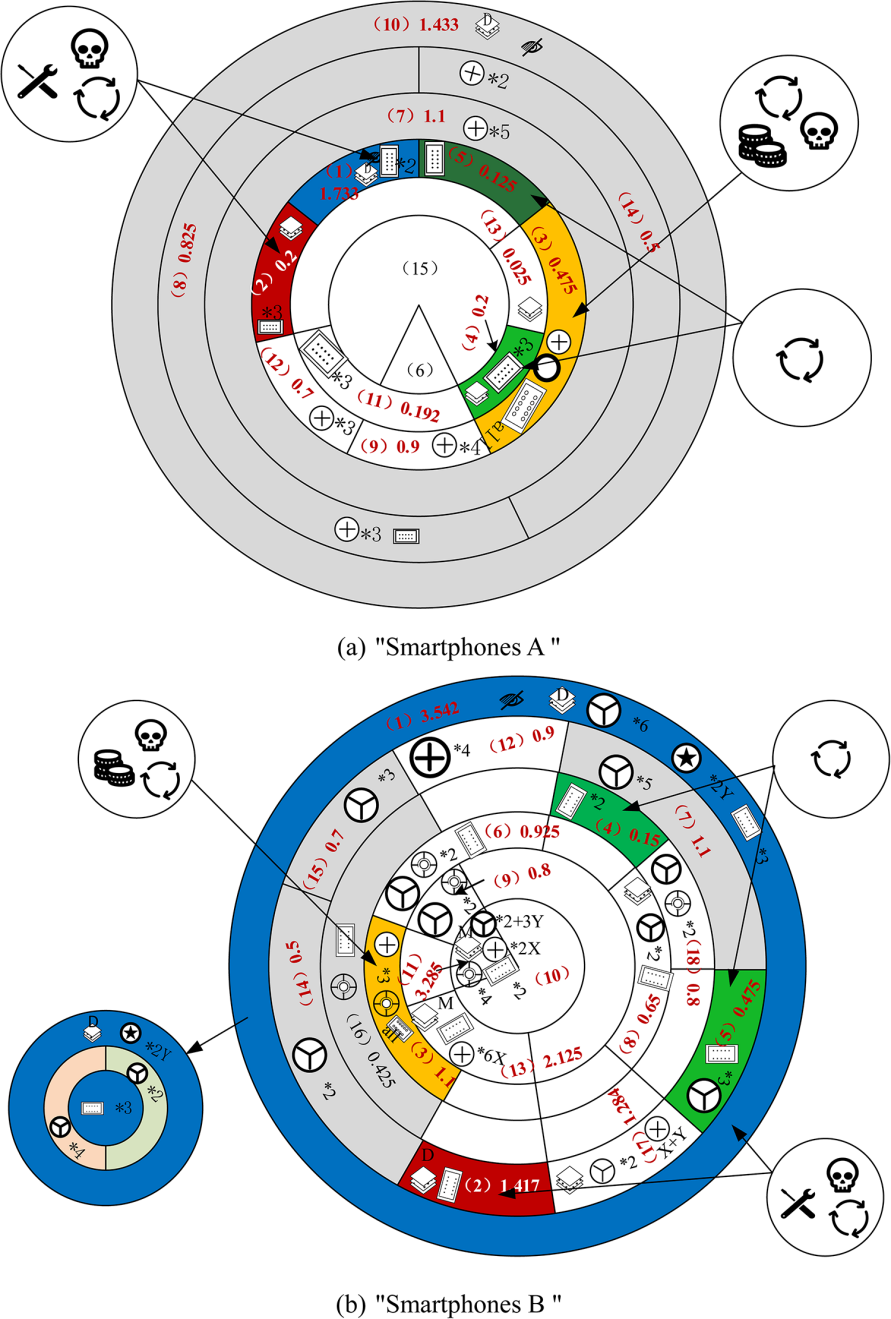
Recycling disassembly schematic. (**a**) “Smartphones A”. (**b**) “Smartphones B”.

Dismantling the main recycling parts of a smartphone, as illustrated in Fig. 8, involves varying levels of difficulty. The five key parts highlighted in color represent primary dismantling challenges. Removing these target components requires first disassembling all other parts depicted in gray, which adds to the overall dismantling complexity.

Table 17 illustrates the recycling and dismantling difficulties, with efficiencies measured as the percentage of target parts recycled and dismantled relative to the total required: 71% for the “Smartphones B” and 41% for the “Smartphones A”. Although the recycling and disassembly efficiency of the “Smartphones B” surpasses that of the “Smartphones A”, the total recycling difficulty for Apple is greater compared to “Smartphones A”.

**Table 17:** Analysis of dismantling difficulty of recycling.

Smartphones model	Difficulty of disassembling target parts	Additional disassembly difficulty	Total	Disassembly efficiency of recycling
“Smartphones A”	2.733	3.858	6.591	41%
“Smartphones B”	6.684	2.725	9.409	71%

For the “Smartphones B”, Fig. 8(b) illustrates the distribution of critical part tiers. Layers 1, 3, and 4 contain 1 part each, while layer 2 contains 2 parts, demonstrating an even distribution across layers. Parts that are challenging to disassemble are found in smaller layers, and constrained parts of the target disassembly have lower disassembly difficulty.

In contrast, for the “Smartphones A”, target parts are predominantly located in layer 4, with the first three layers containing constrained parts that are more difficult to disassemble, resulting in lower recycling and disassembly efficiency. Nevertheless, “Smartphones A” exhibits a lower total recycling and disassembly difficulty compared to the “Smartphones B”, attributed to fewer types and connections of individual parts.

(3) Optimization recommendations.

To reduce the difficulty of disassembling critical recycling components in “Smartphones B”, one effective strategy is to decrease the number of connectors between the target disassembled component and its associated parts. For instance, halving the original 25 screws can lower the total recycling disassembly difficulty to 6.5, similar to “Smartphones A”’s total disassembly challenge. Parts deeper in the hierarchy that are not directly linked to the target part, such as parts 11 and 13, may be excluded from optimization considerations due to their greater disassembly complexity.

“Smartphones A” can enhance recycling and disassembly efficiency by reducing the number of layers containing target parts. Through a redesign that reduces the layers of constrained parts by two, the total recycling and dismantling difficulty can decrease by 2.425 units, leading to a dismantling efficiency increase to 65%.

Whether optimizing key component disassembly during maintenance or recycling processes, designers should prioritize key component optimization to streamline redesign efforts. This approach enhances design efficiency and the practicality of solutions.

## Conclusions

The goal of this study is to provide designers with a visualization model that serves as a foundation and reference for improving product disassembly design. The model not only provides a visual display of component hierarchy priority constraint relationships, but also provides rich disassembly information. Through the Peony diagram model product designers can intuitively and quickly determine the component hierarchy and the shortest disassembly path without constructing huge matrices or adopting complex algorithms, thus saving evaluation time for designers. The disassembly information provided by the model provides an intuitive basis for improving the design of products that are easy to disassemble. In conclusion, the Peony Diagram Model contributes to the field of product disassembly modeling:

1. The peony diagram model clearly demonstrates the easy-to-disassemble structure of the product in an intuitive visualization way, which enriches the knowledge of the disassembly model.
2. A method of reflecting the priority constraint relationship of parts and disassembly information is developed, which provides designers with an easy-to-disassemble design and optimization tool.
3. Provide designers with multiple perspectives to optimize the difficulty of product disassembly from overall and local perspectives. The whole is the disassembly level and disassembly path where the parts are located, and the local is the disassembly information of individual parts.

The model has a wide range of applicability due to its intuitive and informative nature. Whether product designers, design researchers, engineers or maintenance personnel, they can easily understand the distribution of product parts and their disassembly difficulty with the help of the Peony Diagram model, which is convenient for optimizing the design and improving the maintenance efficiency.

This study also has some limitations: (1) The model evaluates the difficulty of product disassembly from the design stage and considers a relatively limited number of evaluation factors, resulting in a deviation between the evaluation results and the evaluation of actual disassembly; (2) This study is currently in the theoretical stage and has not yet been validated in practical applications.Future research could be conducted in the following areas: (1) Improve the evaluation factors and expand the application scope of the disassembly model; (2) Establish a database of product disassembly information, optimize the evaluation system, and form a standardized scoring system to achieve an accurate evaluation of the difficulty of disassembly of individual products.

## Data Availability

The datasets generated and analyzed during the current study are not publicly available because the project is still ongoing, but they can be obtained from the corresponding author upon reasonable request.
